# Analysis of dyslexia candidate genes in the Raine cohort representing the general Australian population

**DOI:** 10.1111/j.1601-183X.2010.00651.x

**Published:** 2011-03

**Authors:** S Paracchini, Q W Ang, F J Stanley, A P Monaco, C E Pennell, A J O Whitehouse

**Affiliations:** † Wellcome Trust Centre for Human Genetics, University of OxfordOxford, United Kingdom; ‡ School of Women's and Infants' Health, University of Western AustraliaPerth, Western Australia, Australia; § Telethon Institute for Child Health Research, Centre for Child Health Research, University of Western AustraliaPerth, Western Australia, Australia; ¶ Neurocognitive Development Unit, School of Psychology, University of Western AustraliaPerth, Western Australia, Australia

**Keywords:** Association study, dyslexia, *DYX1C1*, Raine study, reading skills

## Abstract

Several genes have been suggested as dyslexia candidates. Some of these candidate genes have been recently shown to be associated with literacy measures in sample cohorts derived from the general population. Here, we have conducted an association study in a novel sample derived from the Australian population (the Raine cohort) to further investigate the role of dyslexia candidate genes. We analysed markers, previously reported to be associated with dyslexia, located within the *MRPL19/C2ORF3, KIAA0319, DCDC2* and *DYX1C1* genes in a sample of 520 individuals and tested them for association with reading and spelling measures. Association signals were detected for several single nucleotide polymorphisms (SNPs) within *DYX1C1* with both the reading and spelling tests. The high linkage disequilibrium (LD) we observed across the *DYX1C1* gene suggests that the association signal might not be refined by further genetic mapping.

Dyslexia (reading disability) is a developmental condition with a prevalence ranging from 5% to 17% in school-aged children (Pennington 1990; Shaywitz 1998; Shaywitz *et al.* 1990). As for other neurodevelopmental disorders, initial reports indicated a higher prevalence in males (Finucci & Childs 1981); however, subsequent studies have reported no gender differences (DeFries & Alarcón 1996; Guerin *et al.* 1993). Some studies have suggested that the higher male prevalence could be explained by a referral bias (Vogel 1990) or by greater variance of reading skills in males (Hawke *et al.* 2009).

A strong genetic component for dyslexia has been widely documented (Fisher & DeFries 2002; Habib 2000) and molecular genetic analyses have led to the identification of several candidate genes (Scerri & Schulte-Korne 2010). These include the *MRPL19/C2ORF3* locus on chromosome 2, *ROBO1* on chromosome 3, *KIAA0319* and *DCDC2* on chromosome 6 and *DYX1C1* on chromosome 15. For all of these genes, with the exception of *ROBO1*, associations with common single nucleotide polymorphisms (SNPs) have been reported suggesting that the genetic variants conferring susceptibility to dyslexia are found in the general population. *ROBO1* was identified by the refinement of a breakpoint translocation in one individual with dyslexia and the identification of a rare haplotype co-segregating with dyslexia in a large Finnish family (Hannula-Jouppi *et al.* 2005). So far, support for *ROBO1* has not been reported in additional samples, suggesting its role in the etiology of dyslexia might be restricted to isolated cases. For the other genes, evidence of associations has been described in at least two independent samples.

*DYX1C1* (dyslexia susceptibility 1 candidate 1) was the first candidate gene for dyslexia susceptibility to be identified (Taipale *et al.* 2003). The identification of the gene was initially led by the breakpoint mapping of a chromosome translocation co-segregating with dyslexia in one family. Association analysis in a larger sample supported these findings and implicated two variants in *DYX1C1*, with a putative functional effect. The -3A variant (at the -3G>A or rs3743205 SNP) has a putative effect of transcription regulation and the 1249T variant (at the 1249G>T or rs57809907 SNP) introduces a premature stop codon. These initial associations have been followed by the largest number of replication studies conducted so far for dyslexia candidate genes, with the majority of studies targeting these two specific SNPs. Nine independent studies have analysed rs3743205 and rs57809907 producing conflicting results (Scerri & Schulte-Korne 2010), where association was not detected (Bates *et al.* in press; Bellini *et al.* 2005; Cope *et al.* 2005b; Meng *et al.* 2005a), association was detected with the opposite alleles (-3G or 1249G) (Brkanac *et al.* 2007; Dahdouh *et al.* 2009; Scerri *et al.* 2004; Wigg *et al.* 2004) or association was detected with the same alleles as in the original study (Marino *et al.* 2007). Analysis of allele-specific effect on gene regulation has suggested a functional role for rs3743205 as well as for two other *DYX1C1* markers (rs16787 and rs12899331) (Tapia-Paez *et al.* 2008). Associations with other markers have also been reported for rs3743204, rs685935 and rs17819126 using quantitative reading-related measures in a twin-based Australian sample representing the general population (Bates *et al.* in press). The rs17819126 marker, which was the most significant finding of that study, is a nonsynonymous coding SNP and was associated with three different traits. However, the same SNP, previously called 271G>A, did not yield significant associations in previous studies (Scerri *et al.* 2004; Taipale *et al.* 2003). The rs3743204 marker has also been reported to be associated with dyslexia but only as part of different haplotypes (Dahdouh *et al.* 2009; Wigg *et al.* 2004).

Taken together, association studies of *DYX1C1* have generated contradictory findings based on modest association signals. It has been suggested that differences in population ethnicity or stratification effects could explain conflicting association (Paracchini *et al.* 2007) (Table S1). Indeed, the first association was reported in a Finnish sample, which is a genetic isolate and expected to have a different linkage disequilibrium (LD) landscape. Replications studies have been carried out in samples that varied not only in ethnic origin but also in structure (nuclear families, trios and cases) and ascertainment criteria. However, associations have been consistently reported in independent samples supporting the role of this gene in the development of dyslexia and suggesting that additional efforts are required to elucidate the meaning of these results.

Greater consensus has been reached on the allelic trend of associations for markers within the *KIAA0319* and *DCDC2* genes at the chromosome 6 locus; however, negative replications have also been reported. Both genes have been analysed by testing for association markers distributed all along their genomic sequence in several independent samples (Table S1). Most of the positive associations for *KIAA0319* cluster around the first intron and regulatory sequences upstream of the 5′ end of the gene (Cope *et al.* 2005a; Deffenbacher *et al.* 2004; Dennis *et al.* 2009; Francks *et al.* 2004; Harold *et al.* 2006; Kaplan *et al.* 2002). In particular, rs4504469 which is a coding SNP showed association in independent studies (Cope *et al.* 2005a; Francks *et al.* 2004). This marker is also part of a specific haplotype, effectively tagged by rs2143340, found to be associated in two independent samples selected for severity of phenotype (Francks *et al.* 2004) as well as with reduction in expression of the *KIAA0319* gene (Paracchini *et al.* 2006). The same haplotype was found to be associated with the reading abilities of the general population in two samples representing the general population either with same trend as in the original reports (Paracchini *et al.* 2008) or with opposite direction (Luciano *et al.* 2007). This haplotype has been shown to capture the effect of a common polymorphism, rs9461045, which creates the binding site for a nuclear protein explaining the inhibition of gene expression (Dennis *et al.* 2009). Lack of association at this locus has been reported by studies that instead identified association with the *DCDC2* gene (Meng *et al.* 2005b; Schumacher *et al.* 2006). A functional effect has been proposed for an intronic deletion within *DCDC2* (Meng *et al.* 2005b) but replication attempts have provided only minor support (Brkanac *et al.* 2007; Harold *et al.* 2006; Wilcke *et al.* 2009). Associations have also been reported for several markers including rs807701 (Ludwig *et al.* 2008; Schumacher *et al.* 2006), rs807724 (Meng *et al.* 2005b), rs1087266 (Harold *et al.* 2006; Meng *et al.* 2005b) and rs793862 (Ludwig *et al.* 2008; Meng *et al.* 2005b; Schumacher *et al.* 2006). *DCDC2* has also been investigated in an epidemiological sample, with the most significant association reported for the rs1419228 marker (Lind *et al.* 2010).

Associations at the *MRPL19/C2ORF3* locus on chromosome 2 have been reported in two independent samples (Table S1) with overlapping haplotypes (Anthoni *et al.* 2007). These associations are yet to be replicated in separate studies.

Follow-up studies in epidemiological samples have represented a valid alternative to replicate the original association with dyslexia candidate genes. All these studies have been based on the quantitative analysis of reading-related measures available for individuals representing the general population, regardless of whether a diagnosis of reading impairment was ever conferred (Bates *et al.* in press; Lind *et al.* in press; Luciano *et al.* 2007; Paracchini *et al.* 2008). Besides supporting the roles of these genes in contributing to dyslexia, these investigations have corroborated the notion that dyslexia represents the lower tail of reading abilities, which are normally distributed across the population rather than being a distinct disorder.

Here, we have conducted a replication study by analysing markers previously reported to be associated with dyslexia within the *DYX1C1, KIAA0319, DCDC2* and *MRPL19/C2ORF3* genes in the Western Australian Pregnancy Cohort (Raine) study, which is a longitudinal cohort for which reading-related measures are available. We report association with several markers located within the *DYX1C1* gene.

## Methods

### Participants

The Raine study is a pregnancy cohort that was recruited prior to 18 weeks' gestation from the public antenatal clinic at King Edward Memorial Hospital (KEMH) or surrounding private clinics in Perth, Western Australia (WA) (Newnham *et al.* 1993). Approximately 100 unselected antenatal patients per month were enrolled during this period from August 1989 to April 1992, with a final sample of 2979 women. The inclusion criteria were (1) English language skills sufficient to understand the study demands, (2) an expectation to deliver at KEMH and (3) an intention to remain in WA to enable future follow-up of their child. Participant recruitment and all follow-ups of the study families were approved by the Human Ethics Committee at KEMH and/or Princess Margaret Hospital for Children in Perth. From this original cohort of women, 2868 of their children have been followed over the last two decades with detailed assessments performed every 2–3 years.

### Reading and spelling assessment of children at age 10 years

The Western Australian Literacy and Numeracy Assessment (WALNA) is administered annually to all students across WA in school grades three (age 8), five (age 10) and seven (age 12). The WALNA was developed in consultation with educators to provide information on whether children have reached the minimum standards of reading, writing, spelling and numeracy. The tests have been written to cater for the diverse range of students in Australian schools, and to ensure that there is no systematic bias associated with factors such as gender, culture or geographic location. Every year the WALNA is evaluated by expert judges for content and construct validity and scrutinized by psychometricians for misfitting items, precision and bias. The current study concerned performance on the reading and spelling subtests of the WALNA, completed by the Raine cohort during school grade 5 (between 2000 and 2002).

For the reading test, children were given a magazine and required to answer 33 multiple-choice questions on its contents. A further two questions required a short answer of one to two sentences each. For each item, children were directed to the relevant page and article title (e.g. ‘read *Helicopter* on page 2 of the magazine and answer questions 1–5’). The spelling subtest consisted of two tasks. In the first task, participants were provided with a written paragraph that included 10 spelling mistakes, each of which were circled. The passage was first read aloud by the teacher from beginning to end. The teacher then read through the passage again, this time pausing at each circled word (spelling mistake), upon which children were required to write down the correct spelling of the word. The second spelling task was similar to the first; however, rather than spelling mistakes, the written passage given to children included 14 blank spaces for missing words. The passage, including the missing words, was then read aloud by the teacher twice: the first time, children were instructed to follow the text with their finger, and the second time, children were required to write down each missing word. For items in both the spelling and reading tests, a score (of 1) was awarded for correct answers only. Raw scores for the reading and spelling tests were summed and then converted via a Rasch measurement model (Doig & Groves 2006) into an interval scale to enable easier interpretation of the results. Scores on both the reading and spelling subscales could range from −100 to 800, with higher scores indicating better performance.

These data, collected by the WA Department of Education and Training, were then linked to the Raine study dataset by the WA Data Linkage System using a probabilistic method of matching based on a full name, date of birth, gender and address (Kelman *et al.* 2002). Western Australian Literacy and Numeracy Assessment records were linked for 1038 Raine study children who were in grade five and attending government schools at the time of assessment.

### Genotyping

In the Raine study, DNA was collected using standardized procedures from 74% of adolescence who attended the 14-year follow-up on and a further 5% at the 16-year follow-up. Genome-wide data were generated using an Illumina 660 Quad array for each individual. Only SNPs that passed quality control (QC) criteria (call rate ≥ 95%, minor allele frequency >0.05 and Hardy–Weinberg disequilibrium *P*-value >0.01) were retained for genetic analysis.

### Sample inclusion criteria

Inclusion criteria for the current study were no known intellectual disability; a nonverbal IQ within normal limits as assessed by the Raven's Colored Progressive Matrices (CPM; i.e. a score ≥16th percentile, corresponding to approximately >−1 SD the population average of the 50th centile) and biological parents who were both of white European origin. Furthermore, because the current study had an interest in literacy development of the birth cohort, only those children who spoke English at home were included for analysis.

### Statistical analyses

Our first analysis examined selected SNPs within the *DYX1C1, KIAA0319, DCDC2* and *MRPL19/C2ORF3* genes that have been previously found to be associated with dyslexia. Fourteen of these SNPs were available for the Raine sample (Table S2). These SNPs were tested for association with quantitative measures of reading and spelling scores (Table 1) using an allelic test of association within PLINK version 1.07 (Purcell *et al.* 2007). We also tested for association haplotypes derived from the markers at the *MRPL19/C2ORF3* locus, as previously reported (Anthoni *et al.* 2007). Haplotypes were inferred using SimHap version 1.0.2. In all analyses, gender was specified as a covariate. Principal components analysis with Eigenstrat (Price *et al.* 2006) showed evidence of population stratification and the first two principal components were also included in all analyses.

**Table 1:** Descriptive statistics of phenotypic measures

	*n*	Mean (SD)	Range
Reading (Rasch scale)	520	391.55 (95.42)	−47, 703
Spelling (Rasch scale)	520	422.79 (118.26)	−99, 717
Raven's Colored Progressive Matrices (CPM) (percentiles)	520	62.58	18, 100

The reported *P*-values are not corrected for multiple testing and we show any results with a *P*-value <0.1. The application of a Bonferroni correction would be too conservative because SNPs within the same locus are highly correlated. Instead, we aimed to limit the number of tests by analysing targeted SNPs previously reported in the literature. Any trend of association, even if not significant, would provide additional support for the role of these genes in contributing to dyslexia or reading skills. The LD among *DYX1C1* SNPs in this sample was determined with Haploview version 4.2 (http://www.broadinstitute.org/haploview/haploview) (Barrett *et al.* 2005). Power calculations were computed using Quanto version 4.02 (Gauderman *et al.* 2002) (Fig. S1).

## Results

Literacy data were available for 520 (272 males and 248 females) of the 895 participants who met inclusion criteria (Table 1). All children were turning 10 years of age during the year of WALNA completion. Independent samples *t*-tests and chi-square analyses found that these children differed somewhat to the remainder of the cohort. For example, children who participated in the current study were heavier at birth (participated: M = 3394.55 g, SD = 591.62 g; did not participate: M = 3261.46 g, SD = 625.43 g; *t*(2856) = 4.43, *P* < 0.01) and had mothers who were older at conception (participated: M = 28.39, SD = 5.83; did not participate: M = 27.09 years, SD = 5.91; *t*(2865) = 4.55, *P* < 0.01). Therefore, while there was a modest degree of bias in the current sample favoring more socially advantaged families, the children were not bias toward any pathology.

No significant association was detected for SNPs within *KIAA0319, DCDC2* and *MRPL19/C2ORF3* (data not shown). Haplotype analysis for the *MRPL19/C2ORF3* markers also showed no significant patterns of association. Two SNPs within the *DYX1C1* gene showed a trend of association: rs3743205 (reading: df = 4, *β* = 25.46, *P* = 0.085) and rs685935 (spelling: df = 4, *β* = −12.28, *P* = 0.097). The allelic trend of association for rs3743205, which showed the major allele (or ‘-3G’ allele) associated with poor reading performance, was in agreement with a previous replication study (Wigg *et al.* 2004) reporting opposite trend compared to the original findings (Taipale *et al.* 2003). The minor allele of rs685935 was associated with poor spelling performance, showing the same trend as reported previously with a short-term memory measure (Bates *et al.* in press).

Previous studies of *DYX1C1* have reported associations with a wide range of markers and with opposite allelic trend compared to other dyslexia susceptibility candidate genes. Therefore, while our initial analyses fell short of statistical significance at *P* < 0.05, we followed up the association trend by analysis of all the SNPs available for *DYX1C1* in the Raine sample (Table S3). The SNPs that passed our QC criteria were tested for association with the reading and spelling measures (Table 2). The most significant association (df = 4, *β* = −19.32, *P* = 0.012) was detected between the rs8043049 marker and spelling. This SNP is adjacent to marker rs685935, which was tested in our initial analysis (df = 4, *β* = −12.28, *P* = 0.097) and within a cluster of five SNPs showing a trend of association, including rs8037376 which showed a *P*-value = 0.027 (df = 4, *β* = −17.39). The strongest association with the reading measure was detected with the rs8040756 marker (df = 4, *β* = 18.68, *P* = 0.026).

**Table 2:** Association *P*-values for SNPs in the *DYX1C1* gene

				Reading		Spelling	
					
SNP	Minor allele	Major allele	MAF	*β*	*P*	*β*	*P*
rs8034029	T	C	0.09	—	—	—	—
rs12324434	C	T	0.42	—	—	—	—
rs12594039	C	T	0.06	—	—	—	—
rs7174102	A	T	0.34	—	—	−15.98	0.036
rs3759864	C	T	0.06	—	—	—	—
rs4774768	T	G	0.47	—	—	12.22	0.096
rs7181226	C	T	0.09	—	—	—	—
rs687623	C	T	0.41	—	—	—	—
rs622097	T	C	0.33	—	—	14.35	0.067
rs600753	T	C	0.48	—	—	—	—
rs2290981	A	G	0.06	—	—	—	—
rs7181999	C	T	0.09	—	—	—	—
rs692690	T	C	0.42	—	—	—	—
rs692691	T	C	0.38	—	—	−12.37	0.094
rs7182524	T	C	0.06	—	—	—	—
rs692646	A	G	0.37	—	—	—	—
rs8037376	C	T	0.32	—	—	−17.39	0.027
rs4144134	T	C	0.43	—	—	−12.28	0.097
**rs685935**	**C**	**T**	**0.43**	—	—	−**12.28**	**0.097**
rs8043049	C	T	0.33	—	—	−19.32	0.012
rs6493791	G	A	0.47	—	—	−12.08	0.098
rs16976343	C	T	0.14	—	—	—	—
rs16976349	C	T	0.06	—	—	—	—
rs16976351	C	G	0.07	19.12	0.079	—	—
rs12594443	T	C	0.06	—	—	—	—
**rs17819126**	**T**	**C**	**0.06**	—	—	—	—
**rs3743204**	**T**	**G**	**0.20**	—	—	—	—
**rs3743205**	**A**	**G**	**0.05**	**25.46**	**0.085**	—	—
rs8040756	A	G	0.15	18.68	0.026	—	—

Bolded rows indicate SNPs that previous studies have identified as conferring risk for dyslexia.

MAF, minor allele frequency.

[Correction added after online publication 19 October 2010: in Table 2, major and minor allele were interchanged.]

**Figure 1:**
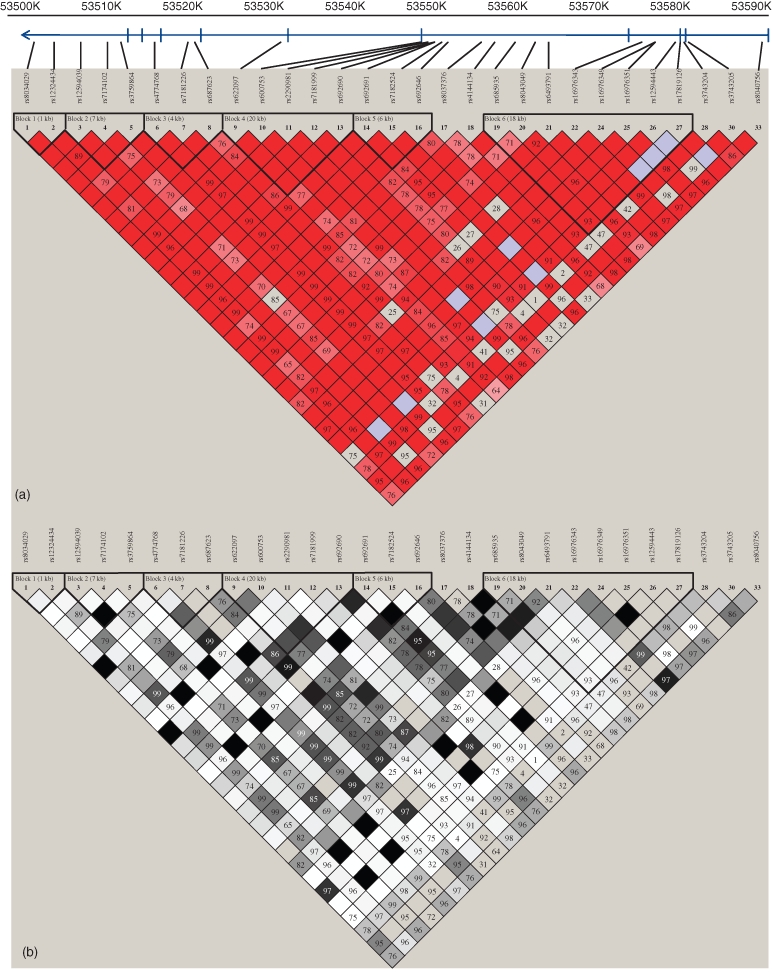
SNPs location and LD across *DYX1C1* The top of the figure shows the structure of the *DYX1C1* gene (blue arrow) indicating the position of exons (blue vertical lines), with an indication of their genomic location on chromosome 15. In total, 29 SNPs were analysed across an 86-kb interval. Black lines indicate the position of each SNP within *DYX1C1*. Inter-SNP LD was generated with Haploview. (a) *D*′ values are reported within cells while empty red cells represent full LD and empty blue cells represent lack of LD. (b) *r*^2^ values are reported within cells where empty white cells represent lack of LD and darker shadings represent increasingly stronger LD. Haploview identified six LD blocks (black solid lines) using the confidence interval method (Gabriel *et al.* 2002), but LD is strong all across the examined region.

Evaluation of intermarker LD showed high LD across the *DYX1C1* locus (Fig. 1). The multiple association signals detected at different markers are most likely a reflection of the high LD background.

## Discussion

In the present study, we have investigated the effect of dyslexia candidate genes on reading and spelling abilities in the Raine sample representing the general population of WA. This is the first study describing a genetic investigation of cognitive traits in this sample, which has so far been used primarily for epidemiological investigations (Chivers *et al.* in press; O'Sullivan *et al.* in press). We detected nominal association signals for several markers within the *DYX1C1* gene, further supporting the role of this gene in contributing to dyslexia and reading abilities more generally. Lack of association with the other genes does not rule out their involvement in dyslexia but their effect cannot be detected in the Raine sample. It is important to note that the reading and spelling WALNA tests used here are different from the tests generally used to ascertain dyslexia. Most of the dyslexia studies are based on test of single word reading or, as in the case of German samples, single word spelling (Schumacher *et al.* 2006). In contrast, the reading test used here is a reading comprehension test and the spelling test is based on the recognition of mistakes. The use of these particular measures combined with a small sample size, yielding limited power to detect genetic associations (Fig. S1), might have prevented the detection of additional genuine associations.

The association *P*-values reported here are weak. However, the association *P*-values reported previously in the literature for *DYX1C1* have been relatively modest, even in samples selected for dyslexia (Paracchini *et al.* 2007). The reason could be that the *DYX1C1* (as well as the other dyslexia candidate genes) effect size is very small. In addition, the discovery samples employed so far have also been of modest size (usually <1000 individuals). Therefore, if any of these genes would contribute to reading abilities in the Raine sample (which has not been selected for dyslexia), we would expect to observe similar levels of association with weak *P*-values.

The role of dyslexia candidate genes have not been completely established, and with the exception of the *KIAA0319* gene (Dennis *et al.* 2009), functional molecular mechanisms have not been described to explain genetic associations. Replication analysis is currently the most valid approach to establish whether these genes indeed contribute to dyslexia and even if association do not reach full statistical significance, association trends may show interesting observation and provide additional evidence.

*DYX1C1* has received the largest number of positive replications of all dyslexia susceptibility candidate genes together with different negative reports. The positive replications have been reported with different SNPs or with opposite alleles of the same SNPs lacking to provide unanimous consensus on the role of this gene. It is important therefore that the present study is considered in the context of these findings. Our data are consistent with previous studies reporting association within *DYX1C1* and, in addition, provide novel elements to interpret of the current body of literature.

We show that in our sample of white European origin, LD is high all across *DYX1C1* and may hinder refinement of the association signal by fine genetic mapping. Therefore, it is not possible to indicate a specific role of coding or regulatory SNPs simply on the basis of genetic associations. These markers, showing relatively stronger *P*-values in some studies (Bates *et al.* in press; Taipale *et al.* 2003), could simply reflect an *a priori* preferential bias in choosing SNPs with potential functional effects but actually acting as proxy of other functional DNA variants in the region. Consistently, our strongest association signals were not observed for markers that have been suggested to have a direct effect on the phenotype, such as rs3743205, rs57809907 (Taipale *et al.* 2003) or rs17819126 (Bates *et al.* in press). Sampling effects and missing data can also lead to differences in association patterns when markers are highly correlated and sample sizes are not particularly large. Our interpretation of the data available so far is that they are suggestive of at least one genuine functional variant within the *DYX1C1 locus*, the effect of which has been picked up by different genetic markers, all in high LD, depending on differences in the samples analysed. Therefore, caution should be applied when interpreting association with markers with a putative functional effect.

*DYX1C1* is an attractive candidate for dyslexia, given its proposed role in neuronal migration and brain development (Wang *et al.* 2006). Neuronal migration has been indicated as a possible biological mechanism underlying dyslexia by neuroanatomical evidences (Galaburda *et al.* 1985). More recently, *DYX1C1* has been shown to interact with estrogen receptors (Massinen *et al.* 2009). The estrogen pathway has been shown to be important during brain development (Wang *et al.* 2003).

In conclusion, our findings are consistent with previous reports in supporting the role of *DYX1C1* in the etiology of dyslexia and modulating reading abilities. Our data suggest that additional work is needed to identify functional genetic variants relevant to dyslexia and to fully understand the function of this gene. However, the high LD across the gene may prevent further refinement of the association by genetic mapping and alternative strategies should be adopted.

## Supporting Information

**Figure S1:** Power analysis. Computed statistical power for the reading (a) and spelling (b) analyses, given a certain *β*-coefficient and minor allele frequency (MAF) and assuming a sample size of 500 participants.

**Table S1:** Summary of sample characteristics reported in the literature

**Table S2:** Descriptive information of SNPs previously reported in the literature to be associated with dyslexia [Correction added after online publication 19 October 2010: Table S2 has been resupplied.]

**Table S3:** Descriptive information of SNPs in the *DYX1C1 gene*

As a service to our authors and readers, this journal provides supporting information supplied by the authors. Such materials are peer-reviewed and may be re-organized for online delivery, but are not copy-edited or typeset. Technical support issues arising from supporting information (other than missing files) should be addressed to the authors.
