# Supplementary material for: Analysis of dyslexia candidate genes in the Raine cohort representing the general Australian population
Source: Genes Brain Behav. 2011 Mar;10(2):158–65. doi: 10.1111/j.1601-183X.2010.00651.x (PMC3084500; doi:10.1111/j.1601-183X.2010.00651.x)
Supplement: Supplementary file 1 [file gbb0010-0158-SD1.doc]

Table S1. Summary of samples characteristics reported in the literature

| Genes | Chr | Reference | Ethnicity | Sample size | Proband affection status^a^ | |
| --- | --- | --- | --- | --- | --- | --- |
|
| *MRPL19/C2ORF3* | 2p11.2 | Anthoni et al. 2007 | Finnish German | 19 families  251 families | Dyslexics Dyslexics |  |
|  |  |  |  |
| *DCDC2* | 6p22.3 | Meng et al., 2005b | US | 153 families | Dyslexics |  |
|  |  | Schumacher et al., 2006 | German | 376 trios | Dyslexics |  |
|  |  | Harold et al., 2006 | UK | 350 cases | Dyslexics |  |
|  |  |  | UK | 143 trios | Dyslexics |  |
|  |  |  | UK | 264 families | Dyslexics |  |
|  |  | Brkanac et al. 2007 | US | 191 trios | Dyslexics |  |
|  |  | Ludwig et al., 2008 | German | 396 trios | Dyslexics |  |
|  |  | Wilcke et al., 2009 | German | 72 cases | Dyslexics |  |
|  |  | Lind et al., 2010 | Australian | 522 twin-families | Unselected |  |
| *KIAA0319* | 6p22.3 | Francks et al., 2004 | UK | 264 families | Dyslexics |  |
|  |  | Cope et al., 2005a | UK | 350 cases | Dyslexics |  |
|  |  |  | UK | 143 trios | Dyslexics |  |
|  |  | Harold et al., 2006 | UK | 350 cases | Dyslexics |  |
|  |  |  | UK | 143 trios | Dyslexics |  |
|  |  |  | UK | 264 families | Dyslexics |  |
|  |  | Dennis et al., 2009 | UK | 264 families | Dyslexics |  |
|  |  | Paracchini et al., 2008 | UK | ~6,000 individuals | Unselcted |  |
|  |  | Luciano et al., 2007 | UK | 440 twin-families | Unselected |  |
| *DYX1C1* | 15q21.3 | Taipale et al., 2003 | Finnish | 109 cases | Dyslexics |  |
|  |  | Scerri et a., 2004 | British | 264 families | Dyslexics |  |
|  |  | Wigg et al., 2004 | Canadian | 148 families | Dyslexics |  |
|  |  | Cope et al., 2005b | UK | 247 trios | Dyslexics |  |
|  |  | Marino et al., | Italian | 212 families | Dyslexics |  |
|  |  | Meng et al., 2005a | US | 150 families | Dyslexics |  |
|  |  | Bellini et al., 2005 | Italian | 57 cases | Dyslexics |  |
|  |  | Brkanac et al. 2007 | US | 191 trios | Dyslexics |  |
|  |  | Dahdouh et al., 2009 | German | 366 trios | Dyslexics |  |
|  |  | Bates et., 2009 | Australian | 789 twin-families | Unselected |  |

^a^ Dyslexia was defined with different criteria across the reported studies

*Table S2 Descriptive information of SNPs previously reported in the literature to be associated with dyslexia*

| Genes | Chromosome | SNP | MinorAllele | Major Allele | MAF | Genotype | HWE  P-value | %  Geno | N | Pass QC | Reference |
| --- | --- | --- | --- | --- | --- | --- | --- | --- | --- | --- | --- |
| C2ORF3 | 2p12 | rs917235 | G | A | 0.451 | 113/253/153 | 0.559 | 99.7 | 519 | Yes | Anthoni et al. 2007 |
| C2ORF3 | 2p12 | rs714939 | A | G | 0.395 | 87/245/181 | 0.249 | 99.1 | 513 | Yes | Anthoni et al. 2007 |
| C2ORF3 | 2p12 | rs730148 | C | G | 0.382 | 68/245/206 | 0.951 | 99.7 | 519 | Yes | Anthoni et al. 2007 |
| DCDC2 | 6p22.3 | rs1419228 | G | A | 0.179 | 20/153/347 | 0.376 | 99.9 | 520 | Yes | Lind et al., 2010 |
| DCDC2 | 6p22.3 | rs793862 | No genotype available | | | | | | | | Meng et al., 2005b  Schumacher et al., 2006  Ludwig et al., 2008 |
| DCDC2 | 6p22.3 | rs807701 | G | A | 0.336 | 56/229/234 | 0.517 | 99.7 | 519 | Yes | Schumacher et al., 2006  Ludwig et al., 2008 |
| DCDC2 | 6p22.3 | rs807724 | C | T | 0.207 | 26/174/309 | 0.329 | 98.4 | 509 | Yes | Meng et al., 2005b |
| DCDC2 | 6p22.3 | rs1087266 | No genotype available | | | | | |  |  | Meng et al., 2005b |
| KIAA0319 | 6p22.3 | rs4504469 | T | C | 0.384 | 81/234/181 | 0.852 | 95.7 | 496 | Yes | Francks et al., 2004  Cope et al., 2005a |
| KIAA0319 | 6p22.3 | rs2038137 | No genotype available | | | | |  |  |  | Francks et al., 2004 |
| KIAA0319 | 6p22.3 | rs6935076 | No genotype available | | | | | | |  | Cope et al., 2005a |
| KIAA0319 | 6p22.3 | rs761100 | A | C | 0.425 | 105/256/158 | 0.636 | 99.7 | 519 | Yes | Harold et al., 2006 |
| KIAA0319 | 6p22.3 | rs9461045 | T | C | 0.206 | 15/158/347 | 0.537 | 99.9 | 520 | Yes | Dennis et al., 2009 |
| KIAA0319 | 6p22.3 | rs2143340 | G | A | 0.157 | 8/139/373 | 0.383 | 99.9 | 520 | Yes | Francks et al., 2004  Paracchini et al., 2008 |
| DYX1C1 | 15q21.3 | rs57809907 | No genotype available | | | | | | |  | Taipale et al., 2003  Scerri et a., 2004 |
| DYX1C1 | 15q21.3 | rs685935 | C | T | 0.431 | 97/257/166 | 0.289 | 99.9 | 520 | Yes | Bates et al., 2009 |
| DYX1C1 | 15q21.3 | rs17819126 | T | C | 0.07 | 2/67/448 | 0.614 | 99.7 | 517 | Yes | Bates et., 2009 |
| DYX1C1 | 15q21.3 | rs3743204 | T | G | 0.197 | 25/154/339 | 0.172 | 99.8 | 518 | Yes | Bates et al., 2009 |
| DYX1C1 | 15q21.3 | rs3743205 | A | G | 0.045 | 2/38/449 | 0.024 | 95.1 | 489 | Yes | Taipale et al., 2003  Wigg et al., 2004 |

MAF = minor allele frequency. HWE P-value is the significance of the Hardy-Weinberg Exact SNP test. %Geno is the genotype success rate across all the individuals genotyped. N is the number of individuals passing our filtering criteria for which genotype data were available.

*Table S3 Descriptive information for SNPs in the DYX1C1 gene.*

| SNP | Minor Allele | Major Allele | MAF | Genotype | HWE P-value | %Geno | N | Pass QC |
| --- | --- | --- | --- | --- | --- | --- | --- | --- |
| rs8034029 | T | C | 0.09 | 7/82/431 | 0.105 | 99.9 | 520 | Yes |
| rs12324434 | C | T | 0.42 | 90/233/196 | 0.953 | 99.7 | 519 | Yes |
| rs12594039 | C | T | 0.06 | 1/52/467 | 0.303 | 99.8 | 520 | Yes |
| rs7174102 | A | T | 0.34 | 67/226/218 | 0.438 | 98.2 | 511 | Yes |
| rs3759864 | C | T | 0.06 | 1/52/467 | 0.303 | 99.8 | 520 | Yes |
| rs4774768 | T | G | 0.47 | 118/252/142 | 0.381 | 98.4 | 512 | Yes |
| rs7181226 | C | T | 0.09 | 7/82/431 | 0.108 | 100 | 520 | Yes |
| rs687623 | C | T | 0.41 | 88/251/181 | 0.765 | 100 | 520 | Yes |
| rs622097 | T | C | 0.33 | 57/242/220 | 0.896 | 99.4 | 519 | Yes |
| rs600753 | T | C | 0.48 | 125/259/136 | 0.355 | 100 | 520 | Yes |
| rs2290981 | A | G | 0.06 | 57/242/220 | 0.435 | 100 | 520 | Yes |
| rs7181999 | C | T | 0.09 | 7/82/431 | 0.108 | 99.9 | 520 | Yes |
| rs692690 | T | C | 0.42 | 100/253/166 | 0.594 | 99.9 | 519 | Yes |
| rs692691 | T | C | 0.38 | 81/239/197 | 0.421 | 99.2 | 517 | Yes |
| rs7182524 | T | C | 0.06 | 1/53/465 | 0.311 | 99.7 | 519 | Yes |
| rs692646 | A | G | 0.37 | 75/241/201 | 0.381 | 99 | 517 | Yes |
| rs8037376 | C | T | 0.32 | 52/228/240 | 0.739 | 99.7 | 520 | Yes |
| rs4144134 | T | C | 0.43 | 97/257/166 | 0.289 | 99.9 | 520 | Yes |
| **rs685935** | **C** | **T** | 0.43 | **97/257/166** | **0.289** | **99.9** | **520** | **Yes** |
| rs8043049 | C | T | 0.33 | 58/225/236 | 0.298 | 99.8 | 519 | Yes |
| rs6493791 | G | A | 0.47 | 112/257/149 | 0.351 | 99.1 | 518 | Yes |
| rs16976343 | C | T | 0.14 | 10/112/394 | 0.107 | 99 | 516 | Yes |
| rs4255730 | C | T | 0.05 | 2/54/415 | 1 | 87.7 | 471 | No |
| rs16976349 | C | T | 0.06 | 1/53/465 | 0.206 | 99.7 | 519 | Yes |
| rs16976351 | C | G | 0.07 | 5/68/447 | 0.033 | 100 | 520 | Yes |
| rs12594443 | T | C | 0.06 | 1/53/464 | 0.212 | 99.7 | 518 | Yes |
| **rs17819126** | **T** | **C** | 0.06 | **2/67/448** | **0.614** | **99.7** | **517** | **Yes** |
| **rs3743204** | **T** | **G** | 0.20 | **25/154/339** | **0.172** | **99.8** | **518** | **Yes** |
| rs12907654 | T | G | 0.02 | 0/21/483 | 1 | 96.5 | 504 | No |
| **rs3743205** | **A** | **G** | 0.05 | **2/38/449** | **0.024** | **95.1** | **489** | **Yes** |
| rs1075938 | T | C | 0.02 | 1/18/496 | 0.359 | 98.9 | 512 | No |
| rs2007494 | A | T | 0.05 | 1/52/466 | 0.05 | 90.2 | 465 | No |
| rs8040756 | A | G | 0.15 | 11/128/380 | 0.050 | 99.9 | 519 | Yes |

MAF = minor allele frequency. HWE P-value is the significance of the Hardy-Weinberg Exact SNP test. %Geno is the genotype success rate across all the individuals genotyped. N is the number of individuals passing our filtering criteria for which genotype data were available. SNPs in bold were analysed in the first step as candidate *DYX1C1* SNPs.

*Figure S1 Power analysis*. Computed statistical power for the reading (*A*) and spelling (*B*) analyses , given a certain beta coefficient and minor allele frequency (MAF) and assuming a sample size of 500 participants.

A)

B)
